# Highlights from 11th Breast-Gynecological and Immunooncology International Cancer Conference (BGICC), 17–18 January 2019, Egypt

**DOI:** 10.3332/ecancer.2019.926

**Published:** 2019-05-01

**Authors:** Hesham El-Ghazaly, Nermean Bahie-eldin, Adel Aref

**Affiliations:** 1 Breast and Gynecological International Cancer Society (BGICS), Cairo 11220, Egypt; 2 Clinical Oncology Department, Ain Shams University, Cairo 11566, Egypt; 3 School of Medicine, University of Adelaide, Adelaide SA 5005, Australia

**Keywords:** breast cancer, gynaecological cancer, immunotherapy, BGICC, BGICS

## Abstract

During the 11th Breast-Gynecological and Immunooncology International Cancer Conference (BGICC), which was held on 17 and 18 January 2019, in Cairo, Egypt, around 100 international, regional and national experts from every continent presented the latest updates in breast cancer, gynaecological cancers and immunotherapy in oncology. Through this report, we highlight the important data and consensus issues that were discussed during the conference.

## Introduction

The Breast-Gynecological and Immunooncology International Cancer Conference (BGICC) annual meeting, which was held on 17 and 18 January 2019 in Cairo, Egypt, was a global, multidisciplinary conference with representatives from 80 nations and every continent. Attendance at the 11th BGICC was increased by 15% and abstract submission was increased by 30% this year. BGICC is now accredited by the European Accreditation Council for Continuing Medical Education.

BGICC has partnered with leading societies of oncology and well recognised cancer foundations worldwide, such as the American Society of Clinical Oncology, the European School of Oncology (ESO), the European Society of Surgical Oncology, the Society of Geriatric Oncology (SIOG), the African Palliative Care Association, the European Society of Radiotherapy, the European Society of Gynaecology, the European Society of Oncology Imaging, the Russian Association of Oncomamoplastic (RAOM), the Faculty of Medicine at Ain Shams Research Institute, the Breast Gynecological International Cancer Society (BGICS), Breast Surgery International, the Society for Immunotherapy of Cancer, the Biobank and Cohort Building Network and Nature research.

The Scientific Programme covered the spectrum of oncology from basic science to palliative care on the treatment of breast and gynaecological cancer, with consideration of pathology, surgery, radiology, radiotherapy, neoadjuvant, adjuvant and metastatic systemic therapy, nursing, clinical pharmacy, palliative care, scientific research, as well as genetics, molecular biology of breast cancer and industry-sponsored symposia. Clinical research was most cited by the participants as their primary topic of interest. In view of personalised medicine, there was an intensive course about Molecular Biology followed by a post-conference three-day ‘hands-on’ workshop in collaboration with Thermofisher and Fudan University concentrating on first Next Generation Sequencing.

## Highlights from the 11th BGICC sessions

### Metastatic breast cancer (MBC)

#### Advances in endocrine therapy

Professor Debu Tripathy (MD Anderson Cancer Center, USA) showed that Ribociclib plus endocrine therapy improved progression-free survival (PFS) compared to placebo plus endocrine therapy and had a manageable safety profile in patients with premenopausal, hormonal positive, human epidermal growth factor receptor 2 (HER2)-negative advanced breast cancer. The combination showed a median PFS of 23.8 months (95% confidence interval (CI) [19.2–not reached]) in the Ribociclib group compared to 13 months (95% CI [11.0–16.4]) in the placebo group (hazard ratio 0.55, 95% CI 0.44–0.69; *p* < 0.001 [1].

Professor Hope Rugo (UCSF Helen Diller Family Comprehensive Cancer Center, USA) discussed the sequence of hormonal treatment in MBC postmenopausal hormonal positive breast cancer patients and how to obtain the maximum benefit for them. Updates from the study Palbociclib plus Letrozole versus Placebo plus Letrozole in Postmenapausal Women with Hormone Receptors Positive/HER2 Negative in Advanced Breast Cancer (PALOMA-2) which explored the efficacy of combining Palbociclib plus Letrozole versus placebo plus Letrozole [2] showed that Palbociclib extended the PFS from 14 to 28 months. Professor Rugo discussed the subset analysis, which showed that Palbociclib was efficient across all the subgroups of the study cohort; however, there was a trend of more benefit in the Luminal-A and B subgroups. Quality of life was maintained in the Palbociclib arm. In addition, she highlighted the toxicity profile of the combination, which should be considered in patient selection. She mentioned that neutropenia grade 1 and grade 2 were the most frequently reported adverse reactions in PALOMA-2 (80%), with 56% and 10% incidence grade 3 and 4 neutropenia respectively.

### Advances in anti-HER2 therapy

Professor Sandra Swain (Washington Cancer Institute, MedStar Washington Hospital Center, Washington, DC, USA) discussed how to individualise anti-HER2 treatment in HER2 positive MBC. The role of immunotherapy in metastatic triple-negative breast cancer (TNBC) without germ-line mutation proved to be effective. Professor Edith Perez (Mayo Clinic College of Medicine, Florida, USA) discussed the data from the IMpassion130 trial which met its co-primary PFS in programmed death-ligand 1 (PD-L1) positive patients, with clinically meaningful overall survival (OS) benefit seen at interim OS analysis in PD-L1 positive patients [3]. Atezolizumab plus nab-Paclitaxel was well tolerated, with a safety profile consistent with each agent. The median overall survival was 25 months with the combination compared to 15.5 months with standard chemotherapy alone. Most side effects were due to chemotherapy and occurred at a similar rate in both treatment groups, although there was a minor increase in nausea and cough in the combination group. Side effects related to immune therapy were rare, the most common being hypothyroidism which occurred in 17.3% of patients receiving the drug combination and 4.3% receiving chemotherapy alone [4].

### Adjuvant treatment

#### Shorter-duration trastuzumab for HER2+ early breast cancer

The optimal duration of adjuvant HER2 therapy has been the subject of multiple, large randomised trials, although there are no compelling data regarding durations of Trastuzumab beyond 1 year. Professor Pierfranco Conte (University of Padova, Italy) discussed the five randomised trials of shorter durations of Trastuzumab which have been conducted. Four of the five shorter-duration trials failed to demonstrate non-inferiority to 1 year of therapy. The fifth trial is a randomised, non-inferiority phase III trial (PERSEPHONE) in which 4,088 women in the UK with HER2 positive early breast cancer received 6 or 12 months of Trastuzumab. Patients also received concurrent chemotherapy or sequential chemotherapy. Patients were stratified by chemotherapy type and timing, trastuzumab timing and estrogen receptor (ER) status (69% were ER+) [5]. Disease-free survival at 4 years (the primary endpoint) was similar between 6 and 12 months of therapy (89.4% and 89.8%, respectively; *p* = 0.01 for non-inferiority), as was overall survival (OS; 93.8% and 94.8%, respectively; *p* = 0.0006 for non-inferiority). In the group receiving 12 months of trastuzumab, 8% stopped treatment because of cardiac toxicity versus 4% of those receiving 6 months of treatment. Professor Pierfranco Conte concluded that some countries with limited resources could benefit from understanding when it is safe to stop administering trastuzumab to subgroups of patients with a low chance of relapse. Professor Conte also discussed the subgroups of breast cancer patients which might benefit from alternative treatment approaches, such as those with node-positive and hormonal positive patients who might get benefit from the addition of neratinib [6].

### Fertility issues in premenopausal breast cancer patients

There is now a clear consensus regarding fertility preservation counselling during adjuvant treatment in premenopausal breast cancer patients. Professor Valentina Guarneri (University of Padova, Italy) explained that breast cancer patients have the lowest chance of becoming pregnant when compared with other cancers; this is mainly due to a risk of toxicity from treatments, as well as the age of the patients at diagnosis. Professor Guarneri emphasised the importance attributed by clinicians to address fertility preservation and the available strategies for preserving fertility in breast cancer patients, including embryo cryopreservation and ovarian tissue cryopreservation preservation in premenopausal breast cancer patients [7].

### Neoadjuvant treatment in breast cancer

Professor Hope Rugo addressed hormonal treatment in luminal breast cancer subtype. She discussed the key outcomes from neoadjuvant hormonal therapy including prognostic biological markers of endocrine sensitivity; a fall in Ki67% of more than 2% to show good prognosis as well as more widespread use of preoperative endocrine prognostic index PEPI score (including progesterone receptor status and lymph node status in order to predict endocrine sensitivity when choosing therapeutic options). Professor Rugo also discussed the duration of neoadjuvant endocrine treatment. While the duration of endocrine treatment in clinical trials is usually standardised at around 3 to 4 months, it was clear that volume reductions continue to occur beyond that duration in a large proportion of cases and routine clinical practice is often to treat to maximum response [8].

Professor Perez discussed the GeparNuevo study which enrolled 174 patients with early TNBC and stratified them for tumour-infiltrating lymphocyte levels: low, 0% to 10%; medium, 11% to 59%; and high ≥ 60%. Patients were randomly assigned to receive Durvalumab or placebo for 2 weeks when a core biopsy was obtained. The experimental arm continued on nanoparticle albumin-bound (nab) paclitaxel plus Durvalumab for 12 weeks, whereas the control arm received nab-paclitaxel alone. Durvalumab added to chemotherapy improved pathologic complete response rates in newly diagnosed TNBC. The best response rates were found when Durvalumab was given for a window of 2 weeks before chemotherapy, priming the immune system first [9].

## BGICC consensus

### Management of advanced cervical cancer

The BGICC international expert panel discussed the best management of advanced cervical cancer. The panel came with consensus about some of the hot topics in the management of advanced and recurrent cervical carcinoma. Some of the important recommendations of the panel were the ‘importance of para-aortic lymph node dissection’ and that it should be included in the routine staging for early cervical cancer in view of the new International Federation of Gynecology and Obstetrics (FIGO) classification. Radical hysterectomy via minimal access surgery should be closely monitored and limited to tumours ≤ 2 cm diameter following a tumour board decision and appropriate patient counselling. For bulky Ib2–Ib3 cervical cancer patients, neoadjuvant chemotherapy plus radical hysterectomy is an acceptable alternative to primary concurrent chemo-radiotherapy.

### Immune-oncology consensus in lung cancer

In 2017, an additional *I* was added to the BGICC to include the new evolving era of immune-oncology. The BGICC started to include special sessions for recent advances in immune-oncology not only in breast and gynaecological tumours but also in different thoracic malignancies. In particular, in terms of precision medicine, an important topic of the conference was ‘BGICS consensus in immune-oncology in lung cancer.’ Thoracic oncology professionals from across the globe gathered together to discuss current issues and the latest research, including future strategies for innovation in this fast-paced field of cancer treatment.

### Management of breast cancer in the elderly

The management of breast cancer in the elderly session was endorsed by BGICS-SIOG-ESO and chaired and moderated by Professor Matti Aapro (Multidisciplinary Oncology Institute, Genolier, Switzerland). The consensus aims to identify optimum radiological imaging, surgical procedures, chemotherapy protocol and prediction of the risk of chemotherapy toxicity in elder patients: The Chemotherapy Risk Assessment Scale for High age patients score. The panel came with very important recommendations which will be published in a separate report.

### Management of axilla in breast cancer

The highlight of the conference was the consensus panel, in which 50 panelists reviewed and discussed the controversies in the management of axilla in breast cancer patients. The goal of this consensus process was to articulate important themes to clinicians around the world in the management of axilla. The panel agreed that the best surgical procedure for breast cancer patients with clinically negative axilla is sentinel lymph node biopsy (SLNB) with its safety during pregnancy and that the best management to avoid axillary dissection in positive axilla is neoadjuvant treatment. The panel recommendations will be published in a separate report.

## Summary

The 11th BGICC which was held in Cairo, Egypt this year summarised the most recent data in the management of breast and gynaecological cancer, as well as in oncological immunotherapy. There were three consensus panel recommendations that covered the most controversial areas in the management of breast and cervical cancer, as well as the management of breast cancer in the elderly. We are expecting to have these recommendations published in separate reports in the near future.
